# Liquid biopsies in precision oncology for older adults with cancer

**DOI:** 10.1038/s41698-026-01285-5

**Published:** 2026-02-05

**Authors:** Yue Zhao, Jaime O. Herrera-Caceres, Jennifer Nobes, ZiCheng Lyu, Marcus Vetter, Claire Falandry, Valentin Goede, Nina Rosa Neuendorff, Kah Poh Loh, Beverly Canin, Hans Wildiers, Nicolò Matteo Luca Battisti

**Affiliations:** 1 Department of General, Visceral, Thoracic and Transplantation Surgery, University Hospital of Cologne, Cologne, Germany; 2 Department of Urology, Penn State Health, Camp Hill, PA USA; 3 Department of Blood Sciences, NHS Tayside & University of Dundee, Ninewells Hospital and Medical School, Dundee, UK; 4 Cancer Center Baselland, Liestal, Switzerland and Medical Faculty University Basel, Liestal, Switzerland; 5 Service de Gériatrie, Centre Hospitaliser Lyon Sud, Hospices Civils de Lyon, Lyon, Pierre-Bénite France; 6 Laboratoire CarMeN, Inserm U1060, INRA U1397, Université Claude Bernard Lyon, Lyon, France; 7 Department of Oncogeriatrics, Center of Geriatric Medicine, St. Marien-Hospital, Cologne, Germany; 8 Department of Geriatrics, Marien Hospital, University Hospital, Ruhr University Bochum, Herne, Germany; 9 Division of Hematology/Oncology, Department of Medicine, Wilmot Cancer Institute, Rochester, NY USA; 10 Cancer and Aging Research Group, City of Hope National Cancer Center, Duarte, CA USA; 11 Department of General Medical Oncology, University Hospitals Leuven, Leuven, Belgium; 12 Department of Medicine, The Royal Marden NHS Foundation Trust, London, UK

**Keywords:** Cancer, Biomarkers, Health care, Oncology

## Abstract

As the global population ages, innovative strategies for cancer management in older adults are urgently needed. Liquid biopsy, a non-invasive tool, offers great promise for this demographic by enabling early detection, real-time monitoring, and therapeutic target identification. This review highlights the clinical utility, feasibility, and safety of liquid biopsies in geriatric oncology, emphasizing their potential integration into routine care to improve treatment outcomes and quality of life for older patients.

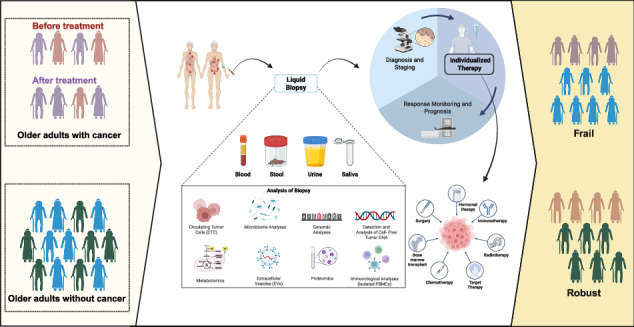

## Introduction

Cancer is predominantly a disease of ageing, with incidence and mortality rates increasing substantially with advancing age^1^. Biological age, which can be assessed by biomarkers, has been shown to be associated with increased risks of various cancers, including lung and colorectal cancers^2^.

As the global population continues to age and the burden of cancer in older adults increases, the need for innovative diagnostic and monitoring strategies tailored to this large cohort becomes more pressing. This demographic shift poses unique challenges, as older adults often present with multiple comorbidities, reduced functional status, and altered physiological responses to cancer and its treatment.

Consequently, there is a growing recognition of the importance of personalized and age-appropriate cancer care that considers the complex interactions between biological, psychological, and social factors in older adults^3^. Increasing evidence suggests an interplay between physiological biomarkers, functional characteristics, social status, and specific comorbidities and the ageing process. Ageing is strongly linked to cancer through several mechanisms: (i) ageing and oncogenesis share common pathways, (ii) ageing tissues can promote tumour progression, and (iii) cancer therapies may accelerate ageing and worsen overall health. Exceptions to this association include paediatric cancers, which primarily occur in infancy^4^, progeroid syndromes, which may not always imply increased risk of malignancy^5^, and the fact that the oldest patients often have lower cancer incidence and mortality rates^4^. Thus, translating biomarkers into clinical practice may require not just biological measures but also a broader assessment of the patient’s overall health status^6^. Liquid biopsies have recently emerged as a promising non-invasive approach for detecting and monitoring cancer. They may also have value for the management of cancer in older adults. Unlike traditional tissue biopsy methods, which may be invasive, costly, and impractical for frail and/or older patients, liquid biopsies offer a minimally invasive alternative providing valuable information about tumour characteristics and dynamics from a simple blood draw or other bodily fluids^7^.

Liquid biopsies encompass techniques for analyzing circulating biomarkers such as cell-free DNA (cfDNA), including circulating tumour DNA (ctDNA, a tumour-derived subset of cfDNA), circulating tumour cells (CTCs), exosomes, microRNAs and metabolomic markers. These analytes collectively reflect the genetic and molecular profiles of tumours and their microenvironment.^8–10^. By capturing the heterogeneity and evolution of tumours over time, liquid biopsies hold promise for early cancer detection, treatment response monitoring, minimal residual disease detection, and prognostic assessment in patients with cancer^11^.

Despite the potential advantages associated with the use of liquid biopsies in older adults with cancer, there are several challenges and limitations that need to be addressed. Age-related changes in circulating biomarker levels, alterations in immune function, and the presence of age-related comorbidities may impact the sensitivity and specificity of this approach in older adults^12,13^. Furthermore, the optimal timing and frequency of liquid biopsy sampling, the choice of biomarkers, and the interpretation of results in the context of age-related physiological changes remain areas of ongoing research and debate. Therefore, there is a critical need for comprehensive evaluation and validation of liquid biopsy technologies in cancer patients of older age to determine their clinical utility, reliability, and cost-effectiveness in real-world clinical settings.

Liquid biopsies may enable better and more rapid treatment decisions for older patients with cancer by providing a better understanding of the biology of the tumour, while geriatric assessment (GA) provides a more accurate insight into the patient’s health status and opportunities to inform geriatric interventions [i.e., comprehensive geriatric Assessment (CGA)]. Therefore, they may be both considered as unique opportunities to provide personalized cancer care.

**Fig. 1:**
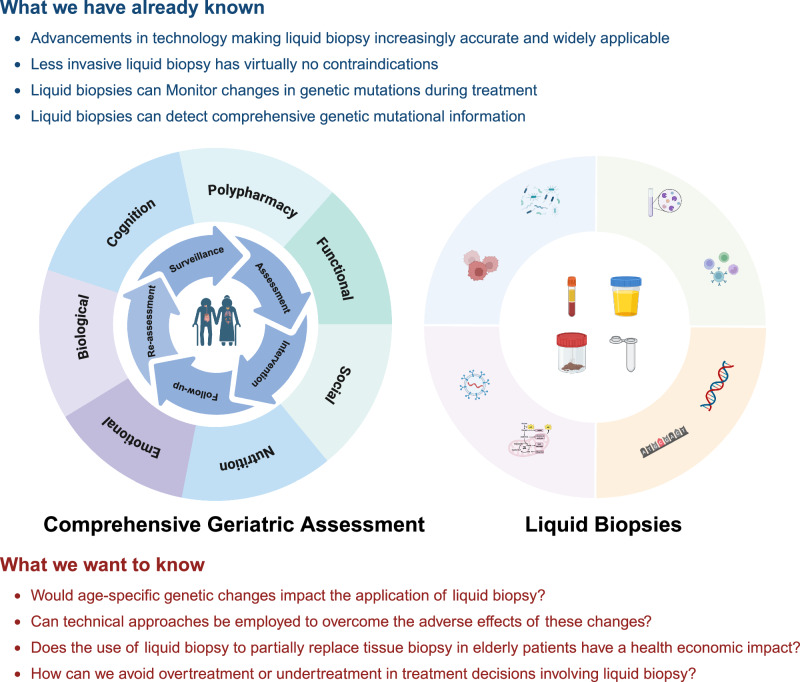
The value and challenges of liquid biopsy as an integral component of assessing older adults with cancer. Liquid biopsy may be integrated as part of the routine evaluation for older adults with cancer, in combination with CGA. We still face several challenges and overcoming them will require more translational research and support from clinical data.

In this comprehensive review, SIOG Translational Research Working Group—comprising geriatricians, medical oncologists, haematologist, surgical oncologists, radiation oncologists, nurses, and allied health professionals as well as a patient advocate—aims to evaluate the current evidence on liquid biopsy applications for older adults with cancer, including their diagnostic accuracy, prognostic value, and clinical applications. This paper also provides insights to identify key research directions to optimize the use of liquid biopsies in this population (Fig. 1).

## Methods

This comprehensive review was conducted to evaluate the current state, benefits, and challenges of liquid biopsy applications in older adults with cancer. A systematic approach was employed following the Preferred Reporting Items for Systematic Reviews and Meta-Analyses (PRISMA 2020) guidelines^14^. Articles were identified relating to the use of liquid biopsies in translational research and clinical trials; these included ctDNA, CTCs, and other approaches such as extracellular vesicles and -omics analysis. The search was performed in both PubMed and Embase to identify articles published in the 15 years preceding November 1, 2024. The search strategy is depicted in the Supplemental Method.

Abstracts were screened by Yue Zhao and Zicheng Lyu prior to review of the full-text articles. The clinical research and clinical translational research on liquid biopsy in tumour diagnosis, staging, treatment, and prognosis were included, while those without age-related information (which can be in the form of subgroups, or mean and standard deviation, or median and range, etc.) were excluded. Studies that only describe detection techniques or are mainly used for laboratory research without describing clinical applications were also excluded.

## Results

**Fig. 2:**
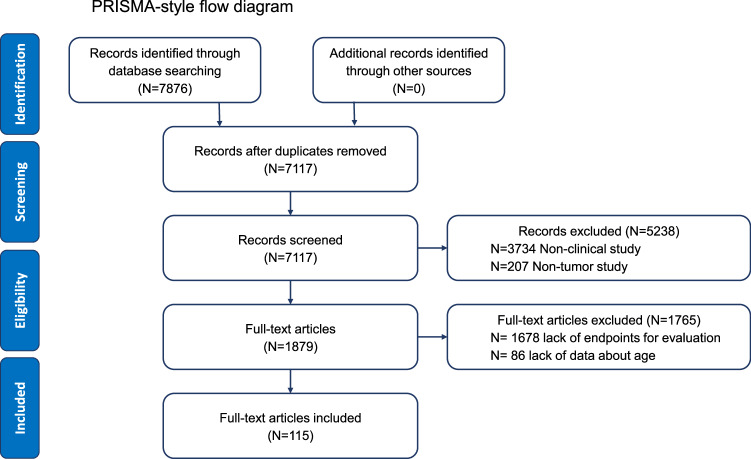
PRISMA-style flow diagram.

Following screening and exclusions, 115 full-text publications were included (Fig. 2). We summarize the findings on the utility of liquid biopsies for early detection and diagnosis, treatment monitoring, and response assessment for older adults with cancer below.

### Potential application of liquid biopsies to cancer diagnosis and staging

Liquid biopsies have potential advantages over traditional, invasive tissue biopsies, making them an attractive option for older adults. As described earlier, liquid biopsies can be performed on a variety of body fluids ranging from those collected non-invasively, such as saliva, urine or faeces, to samples with more invasive collection, such as cerebrospinal or pleural fluids (Table 1).

**Table 1:** Liquid biopsy in tumour diagnosis and staging

Tumour	Patient population	Age group	Number of patients in the corresponding age group	Format	Target	AUC (sensitivity/specificity)	Primary Outcomes	Reference
HCC	466	≤50 >50	322 144	Blood cfDNA mutations	TERT, TP53, CTNNB1, AXIN1	0.93 (100.0/94.0)	Detection	^101^
HCC	55	58 (24–87)	n.a.	Blood cfDNA mutations	APC, ARID1A, AXIN1, BRAF, CDKN2A, CTNNB1, EGFR, KRAS, PIK3CA, PTEN, STK11, TP53, TERT	0.86 (81.0/81.0)	Detection	^102^
HCC	1098	55 (15–81)	n.a.	Blood cfDNA methylation signatures	401 HCC-specific cfDNA methylation markers	0.94 (83.3/90.5)	Early HCC detection; prognostic survival	^103^
HCC	Phase I: 21 patients with HCC Phase II: 95 patients with HCC	64 (57–70)	n.a.	Blood cfDNA methylation signatures	6 HCC-specific cfDNA methylation markers (HOXA1, EMX1, ECE1, AK055957, PFKP, CLEC11A)	Phase I: 0.91 (86.0/87.0) Phase II: 0.96 (95.0/92.0)	Staging	^104^
HCC	Training set: 120 Validation set: 67	53 (24–83)54 (23–74)	n.a.	Blood cfDNA methylation signatures	2321 methylated cfDNA markers	0.93 (84.0/96.0)	Detection	^105^
HCC	156	63 (59–70)	n.a.	Blood cfDNA methylation signatures	3 HCC-specific cfDNA methylation markers (HOXA1, TSPYL5, B3GALT6)	0.86 (88.0/82.0)	Detection	^106^
HCC	122	55	n.a.	Blood cfDNA methylation signatures	28 HCC-specific cfDNA methylation markers	0.94 (75.7/91.2)	Detection	^107^
HCC	504	n.a.	n.a.	Blood cfDNA methylation signatures	Differential methylation of CpGs: CHFR, VASH2, CCNJ, GRID2IP, F12	0.94 (84.5/95.0)	Early HCC detection and Staging	^108^
HCC	158	67.5 (62–73)	n.a.	Blood Extracellular vesicles	EV-derived 10 mRNA signature (AFP, GPC3, ALB, APOH, FABP1, FGB, FGG, AHSG, RBP4, TF)	0.93 (94.4/88.5)	Detection	^109^
HCC	Training set: 106 Validation set: 72	64 (59–70)67 (62–70)	n.a.	Blood Extracellular vesicles	EpCAM+CD63+, CD147+CD63+, and GPC3+CD63+ EVs	0.93 (94.0/81.0)	Detection	^110^
HCC	209	≤60 >60	38(CLD) + 34(HCC) 47(CLD) + 68(HCC)	Blood Extracellular vesicles	Three small RNA clusters	0.87 (86.0/91.0)	Detection	^111^
HCC	124	≤60 >60	35 89	Blood Extracellular vesicles	EV-derived 3 miRNA signature (miR-122-5p, let7d-5p, and miR-425-5p)	0.95 (89.0/92.0)	Detection	^112^
HCC	150	60.8	n.a.	Blood Extracellular vesicles	EV-derived 3 miRNA signature (miR-26a, miR-29c, miR-199a)	0.97 (92.0/90.0)	Detection	^113^
NSCLC	13	≤60 >60	6 7	Blood ctDNA	Blood ctDNA	Stage II–IV 0.99 (100/96) All stages 0.95 (85/96)	NSCLC detection and Staging	^114^
NSCLC	96	≤60 >60	19 77	Blood ctDNA	Blood ctDNA	Sensivity 99% Specificity 99.6%	Detection	^115^
Breast cancer (*n* = 45), colorectal cancer (*n* = 42), lung cancer (*n* = 65), ovarian cancer (*n* = 42), healthy (*n* = 50)	244	≤60 >60	162 82	Blood ctDNA	Blood ctDNA	Sensitivity 97.4% Specificity 89-100%	Detection	^116^
NSCLC	125	n.a.	n.a.	Blood cfDNA methylation signatures	6 cfDNA methylation markers (CDO1, HOXA9, AJAP1, PTGDR, UNCX, and MARCH11)	Sensitivity 72.1% Specificity 71.4%	Detection	^117^
NSCLC	200	71 (45-92)	n.a.	Blood cfDNA methylation signatures	6 cfDNA methylation markers (MGMT,p16INK4a,RASSF1A,DAPK, and RARb)	Sensitivity 49.5% Specificity 80.5%	Detection	^118^
NSCLC	Training set: 104 validation set: 46	70 (42-87) 69 (52-83)	n.a.	Blood ctDNA	Blood ctDNA	AUROC 0.69–0.98 Sensitivity 42–80% Specificity 64–100%	Detection	^31^
50 solid tumours	Training: 3052 validation: 1264	≤65 >65	1386(Control) + 1181(Tumour) 745(Control) + 1004(Tumour)	Blood ctDNA	Blood ctDNA	Sensitivity 44.2–69.8% Specificity 98.3–99.8%	Detection	^119^
7 solid tumours	189	n.a.	n.a.	Blood ctDNA	Blood ctDNA	Sensitivity >95% Specificity 100%	Detection	^120^
PC	80	≤60 >60	37 43	Blood CTCs	CD45–/DAPI+/CEP8 ≥ 3	0.85 (76.0/94.0)	Detection	^121^
PC	45	≤60 >60	12 33	Blood CTCs	CD45–/DAPI+/Folate receptor+	0.837 (86.7/83.3)	Detection	^122^
PC	72	n.a.	n.a.	Blood CTCs	DAPI+/CD45-, and CK+ or CEA+	0.867 (75/96.4)	PC detection and Staging	^123^
PC	68	≤66 >66	34 34	Blood CTCs Blood ctDNA	Blood CTCs Blood ctDNA	CTCs Sensitivity 67% Specificity 80% ctDNA Sensitivity 65% Specificity 75%	Detection	^124^
PC	39	60.1 (29–83)	n.a.	Blood ctDNA methylation signatures	ADAMTS1 and/or BNC1	0.95 (97.4/91.6)	Detection	^125^
PC	95	66 (45–85)	n.a.	Blood ctDNA methylation signatures	BMP3, RASSF1A, BNC1, MESTv2, TFPI2, APC, SFRP1 and SFRP2	Stage I–II 0.86 (73/83) All stages 0.86 (76/83)	PC detection and Staging	^126^
PC	20	≤65 >65	6 14	Blood Extracellular vesicles	lncRNA HEVEPA	0.78 (66.7/78.6)	Detection	^127^
BC	31	n.a.	n.a.	Urine cfDNA mutations	TERT promoter	Sensitivity 67.7% Specificity 88.0%	Detection	^128^
BC	125	63 (15–89)	n.a.	Urine cfDNA mutations	Urine supernatant (TERT, FGFR3, TP53, PIK3CA, and KRAS); urine sediments (TERT, FGFR3, TP53, HRAS, PIK3CA, KRAS, and ERBB2)	Urine supernatant: 0.94 (87/100); Urine sediments: 0.91 (82.6/97)	Detection	^129^
BC	Training: 137 Validation: 79	≤60 >60	44 172	Urine cfDNA methylation signatures	DMRTA2	0.926 (82.9/92.5)	Detection	^130^
BC	147	≤60 >60	17 130	Urine cfDNA methylation signatures	GHSR, MAL	0.89 (92/85)	Detection	^131^
BC	63	72 (64–75)	n.a.	Urine cfDNA methylation signatures	GHSR, MAL	0.87 (79/80)	Detection	^132^
BC	Training: 313 Validation: 175	≤60 >60	212 276	Urine cfDNA methylation signatures	OTX1, SOX1-OT	Sensitivity 90.0% Specificity 83.1%	Detection	^133^
BC	464	n.a.	n.a.	Urine cfDNA methylation signatures	ONECUT2, VIM	AUC 0.898–0.935 Sensitivity 87.1–91.2% Specificity 82.9–89.7%	Detection	^134^
BC	136	≤60 >60	115 21	Urine cfDNA methylation signatures	TRNA-Cys, SIM2, NKX1–1	0.971 (93.5/92.6)	Detection	^135^
BC	252	n.a.	n.a.	Urine cfDNA methylation signatures	PCDH17, POU4F2, PENK	AUC 0.96–0.99 Sensitivity 84–87% Specificity 97–100%	Detection	^136^
BC	192	n.a.	n.a.	Urine cfDNA methylation signatures	HOXA9, PCDH17, POU4F2, and ONECUT2	0.871 (90.5/73.2)	Detection	^137^
Upper tract urothelial carcinoma	42	n.a.	n.a.	Urine cfDNA methylation signatures	GDF15, TMEFF2, VIM	0.923 (91/100)	Detection	^138^
BC	Training: 280 Validation: 120	≤66 >66	208 192	Urine lncRNA	lncRNA UCA201–1, HOTAIR, HYMA1,MALAT95	AUC 0.95 Sensitivity 93.3–95.7% Specificity 94.3–96.7%	Detection	^139^
BC	104	n.a.	n.a.	Urine Extracellular vesicles	miR-93-5p, miR-516a-5p	0.867 (85.2/82.4)	Detection and Staging	^140^
EC	334	≤55 >55	35 299	Blood cfDNA methylation signatures	OTOP2 and KCNA3	0.903 (87.4/93.3)	Detection	^141^
GC	116	60 (33–89)	n.a.	Blood CTCs	DAPI+/CD45–/CK+ cells or DAPI+/CD45–/EpCAM+ cells	0.928 (85.3/90.3)	Detection	^142^
GC	230	≤65 >65	160 70	Blood cfDNA	Multi-dimensional cell-free DNA	AUC 0.937–0.972 Sensitivity 88.2–91.8% Specificity 87.2–92.1%	Detection	^143^
CRC	248	60 (24–89)	n.a.	Blood ctDNA methylation signatures	11 cfDNA methylation markers	0.91 (83.9/85.7)	Detection	^144^
CRC	120	≤65 >65	86 34	Blood cfDNA	Blood cfDNA level	AUC 0.788–0.893	Detection	^145^
CRC	219	≤70 >70	171 48	Blood Extracellular vesicles	FIBG, PDGF-β and TGF-β	AUC 0.882–0.937	Detection	^146^
CRC	398	≤70 >70	300 98	Blood cfDNA	NTMT1 and MAP3K14-AS1	0.918 (91.2/92.4)	Detection	^147^
BTC	40	≤59 >59	20 20	Blood ctDNA methylation signatures	OPCML and HOXD9	0.812 (62.5/100)	Detection	^148^
OSCC OPSCC	230	≤50 >50	11 219	Salivary RNA	Salivary host and microbiome RNA signature	0.96 (85/94)	Detection	^149^
HPV+ oropharyngeal cancer	117	62 (45–93)	n.a.	Blood Circulating tumour HPV DNA	Circulating tumour HPV DNA	Sensitivity 100% Specificity 94.4%	Detection	^150^

The presented clinical studies focus on the role of different types of liquid biopsies in tumour diagnosis and staging, demonstrating their sensitivity, specificity, and AUC. However, older adults were not listed as a separate subgroup.

*HCC* hepatocellular carcinoma, *NSCLC* non-small cell lung cancer, *PC* pancreatic cancer, *BC* bladder cancer, *EC* esophageal cancer, *GC* gastric cancer, *CRC* colorectal cancer, *BTC* biliary tract cancer, *OSCC* oral squamous cell carcinoma, *OPSCC* oropharyngeal squamous cell carcinoma.

However, the most widely used sample type is blood—obtained via the minimally invasive procedure of venipuncture. Venipuncture is associated with lower rates of procedural complications and is generally more acceptable to patients compared with tissue biopsy, especially where repeat sampling may be required^15,16^. The use of blood or other non- or minimally invasive sample types is particularly beneficial in frail and/or patients with comorbidities in whom the risk of adverse outcomes may be higher than when undergoing invasive procedures^17^.

In older patients, liquid biopsies may be used to guide escalation or de-escalation of therapy to avoid over- or under-treatment^18^. Additionally, liquid biopsies may be done at home, which may provide an opportunity to improve access to care for older adults^19^. Despite these potential advantages, it is important to consider the potential impact of age on the analysis and the interpretation of liquid biopsies.

#### cfDNA in blood

Liquid biopsies can be tumour-informed, meaning they are specific to the patient based on prior mutation analysis of tumour tissue, or tumour-agnostic (also known as tumour-naïve or tumour-uninformed), comprising predetermined tumour-associated genetic targets interrogated within cfDNA that are standard across patients^20^. Healthy older adults have higher concentrations of cfDNA due to changes related to the ageing process, such as inflammation, cellular stress, and apoptosis^21^.

The majority of this cfDNA is hematopoietic in origin and may demonstrate mutations associated with clonal hematopoiesis (the clonal expansion of hematopoietic stem cells), which are implicated in haematological malignancies^22,23^. The prevalence of these mutations rises with age, and they have been found to be present in over 90% of tested individuals when ultra-deep sequencing is used^23,24^.

If an underlying haematological malignancy is absent, these mutations are related to clonal haematopoiesis of indeterminate potential (CHIP). In the definition proposed in the latest update of the World Health Organization (WHO) classification of myeloid malignancies^25^, CHIP is formally defined as a somatic clonal mutation in a gene frequently mutated in haematologic malignancies, detectable in peripheral blood at a variant allele frequency of ≥2%, but without clinical features of a haematologic malignancy or another described clonal disorder^26^. Using this definition, more than 10% of adults over the age of 70 years demonstrate CHIP, rising to almost 20% in those over age 90^27^.

CHIP may be a precursor to haematological malignancy, with around 1% of patients progressing to overt cancer each year, much akin to monoclonal gammopathy of uncertain significance (MGUS) as a precursor for myeloma, and has been associated with an increased risk of developing cardiovascular disease^28,29^. The most implicated genes in CHIP include *DNMT3A, TET2* and *ASXL*, and after prior genotoxic treatment approaches, TP53 and PPM1D.

However, there is considerable overlap between genes implicated in CHIP and those involved in solid organ carcinogenesis (such as *TP53, KRAS, NRAS*, and *PIK3CA,* amongst others)^23^. One study of 124 patients with cancer found that over half of all mutations detected in cfDNA were also detected in white blood cells (WBC), demonstrating the potential of CHIP mutations to confound liquid biopsy results, especially when high-depth sequencing is used^24^.

An additional five studies investigating multi-gene cfDNA panels in patients with various solid tumours found the prevalence of clonal hematopoiesis-derived mutations in their liquid biopsies to be between 15-58%^30–36^. Of note, recent research has shown that CHIP prevalence increases after anti-cancer therapy in patients with solid tumours, likely due to genotoxic stress, which should be considered where liquid biopsies are used for post-therapy monitoring^27^.

Older adults are affected in cancer more significantly than their younger counterparts due to increased CHIP prevalence, which can increase the risk of false-positive results and potentially lead to inappropriate or unnecessary therapy in frail patients^37^. Therefore, it may be beneficial to retain the buffy coat during sample processing to allow sequencing of both cfDNA and WBC DNA. Selecting an appropriate read depth for WBC sequencing is crucial to ensure that CHIP mutations with low variant allele frequency are accurately identified^23^. Despite this, many of the currently approved commercial assays involve plasma analysis only^38^. Of note, germline variants will also be evident in WBC DNA but may be recognized by the allele frequency^38^.

In addition, across multiple solid malignancies (including colorectal, lung, breast, biliary tract and head and neck cancer), detectable cfDNA mutations differ based on patients’ age^39–42^ possibly owing to differing pathways of carcinogenesis. Therefore, considering the patient cohort in which a genetic panel was developed is critical to assess its applicability to older adults, and vice versa. Since the incidence of multiple malignancies is generally higher in older adults than in younger populations, multicenter studies of cfDNA-based multicancer early detection (MCED) in asymptomatic middle-aged and older individuals (aged ≥40^43^ or ≥50^44^) are currently underway. Although true-positive cases remain relatively low (0.19–0.53%) and coverage by health insurance is limited^43,44^, these studies provide important high-quality evidence for early cancer screening specifically targeting asymptomatic older adults.

Across different age groups, in both tumour-informed and tumour-agnostic biopsies, it is also important to consider the possibility of false-negative results. These may arise from concentrations of tumour-derived DNA which are below the detectable limit, for reasons such as low sample input volume, low shedding from the tumour, or inadequate analytical sensitivity^45^.

#### CTCs and CTC clusters in blood

CTCs are precursors of metastasis in various solid cancers. Evaluation of CTC levels can be used for tumour metastasis prediction, prognosis evaluation, drug exploitation, and individualized treatment, meaning they exhibit outstanding clinical application prospects. In recent years, accurately capturing and analyzing CTCs has become a research hotspot in the early diagnosis and precise treatment of tumours. However, we are not aware of any specific impacts of ageing on the interpretation of CTC-based liquid biopsies.

CTC clusters, which are aggregates of cancer cells that detach from primary tumours and travel through the bloodstream, exhibit a significantly higher metastatic potential compared to individual CTCs, contributing to cancer progression and poorer patient outcomes^46,47^.

To date, there is no clear evidence that ageing per se significantly affects the interpretation of CTC-based liquid biopsies. However, medications commonly used in older adults may influence CTC dynamics. Recent research has identified the Na⁺/K⁺-ATPase enzyme, which is a target of classic positive inotropic drugs used for patients with heart failure or atrial fibrillation, with the majority of these patients being older adults, as a key player in maintaining the integrity of CTC clusters. Inhibitors of Na⁺/K⁺-ATPase, such as digoxin, have been shown to disrupt these clusters, effectively dissociating them into single cells and blocking metastasis^48,49^. This dissociation leads to the reversal of epigenetic changes associated with cluster formation, thereby reducing the clusters’ metastatic potential^50^. Thus, CTC clusters play a critical role in cancer metastasis, and their dissolution via Na⁺/K⁺-ATPase inhibition represents a promising strategy to limit metastatic spread and improve patient outcomes, potentially across all subgroups, including older and frail patients.

#### Other liquid biopsy types utilizing blood samples

Circulating RNAs (including small non-coding RNA, micro-RNA, and long non-coding RNA) and exosomes are released dynamically from cancer and non-cancer tissues and age is associated with increased release^51–53^. Liquid biopsy studies involving RNA or exosome analysis therefore require age-matched controls and age-related interpretation.

The same is true for metabolomic biomarkers for cancer, which are influenced by age, sex, nutrition, and diet^54,55^. As these technologies continue to be improved, further research into their suitability for use in older adults will be required.

#### Considerations when using other types of liquid biopsy sampling

There is a paucity of information regarding specific considerations for older adults when utilizing other sample types. However, some of the considerations above will also apply to different specimens; for example, age-related changes in the metabolome are also evident in saliva, urine, cerebrospinal fluid, and faeces^56–59^.

In recent years, a number of studies have been published attesting to the significant role of liquid biopsies (including urine cfDNA, urine long non-coding RNA [lncRNA], and urine extracellular vesicles) in the diagnosis and staging of urological malignancies, such as upper tract urothelial carcinoma, bladder cancer, and prostate cancer (Table 1), driven by the fact that the procurement of urine is markedly more facile and entirely non-invasive compared to that of blood.

Using other types of liquid biopsy specimens in older adults remains challenging, both at the sampling and analytical stages. In the case of saliva, sample acquisition can be affected by the slower salivation rate associated with aging and the use of certain medications, which may compromise sample quantity and/or quality^60^. Similarly, age-related changes in urinary function, including increased frequency, urgency, and incomplete emptying, may affect the ease and reliability of urine sample collection^61^. Furthermore, although non-invasive sampling offers potential benefits for older adults, many of these specimen types rely on self-sampling techniques. Therefore, collection devices and associated instructions should be designed in a format accessible to older adults, who are more likely to experience lower health literacy as well as functional or cognitive impairments compared with younger individuals^62^. In summary, adapting pre-analytical protocols for this demographic is essential to ensure reliability and accuracy across specimen types.

At the analytical stage, urine-based liquid biopsies face additional challenges, as bacterial growth can cause tumour DNA to be overwhelmed by bacterial DNA^63^. Older adults, particularly older women, are more likely to have bacteriuria (either asymptomatically or with evidence of urinary tract infection)^64,65^ and are therefore disproportionately affected, which may impact the validity of the liquid biopsy result^66^.

### Potential applications of liquid biopsies to personalize therapy decision-making

The identification of genetic and molecular tumour characteristics has revolutionized the decision-making process for treatment selection. Precision oncology, informed by biopsy-based pathology, extends beyond mere diagnosis and staging to encompass the determination of molecular subtypes and the identification of therapeutic targets.

Current standard practice for patients with advanced cancer increasingly relies on tumour molecular profiling to identify those with oncogene-addicted diseases. For instance, genomic testing enables the selection of appropriate targeted treatment options able to improve treatment outcomes and the prognosis of patients with non-small cell lung cancer (NSCLC), which is prevalent in older adults.

On a global scale, biomarker-guided therapy with novel agents has achieved response rates, such as osimertinib in EGFR-mutant NSCLC (80%), alectinib in ALK-positive NSCLC (83%), and entrectinib (77%) and crizotinib (72%) in ROS1 fusion-positive NSCLC^67–69^.

Beyond disease-specific biomarkers, pan-cancer biomarkers have been identified, including microsatellite instability (MSI) and high tumour mutational burden (TMB), which serve as tissue-agnostic predictive markers for immunotherapy response. Pembrolizumab and nivolumab are FDA-approved immunotherapeutic agents for tumours with high MSI or deficient mismatch repair (dMMR).

Compared with younger patients, older patients may face specific challenges in cancer treatment: they often have more frequent contraindications to surgery and anaesthesia due to comorbidities, poorer physical performance, or the use of antiplatelet or anticoagulant medications, making systemic therapy increasingly important. Additionally, they may have a lower tolerance to the adverse effects of systemic therapy, necessitating higher precision in the selection of systemic treatment regimens. For older patients not suitable for tissue biopsies, clinicians may utilize liquid biopsies to obtain valuable genetic expression or mutation information, thereby enabling the delivery of personalized precision therapy (Table 2). However, molecular profiling alone does not capture the full spectrum of age-related vulnerabilities. Integrating molecular profiling with CGA allows clinicians to tailor treatment intensity based not only on tumour-specific genetic alterations but also on the patient’s functional status, comorbidities, cognitive function, and frailty. This combined approach can help select the most appropriate systemic therapy, adjust dosing to minimize toxicity, prioritize supportive care interventions, and ultimately improve treatment efficacy and quality of life in older adults (Fig. 1).

**Table 2:** Applications of Liquid biopsies for specific treatment options

Tumour	Patient population	Age group	Number of patients in the corresponding age group	Format	Target	Applied treatment	Main Results	Reference
PC	100	64 (52–73)	n.a.	Blood CTCs	Vimentin-positive CTCs	Chemotherapy	Significantly reduced CTC counts were observed after chemotherapy in subjects that responded to treatment (*n* = 15)	^151^
PC	136	68 (59–74)	n.a.	Blood CTCs	eCTCs: CK+, vimentin-, CD- mCTCs: CK+, vimentin+, CD-	Chemotherapy	Increased CTCs counts were observed in early recurrence: 0.871 (82.0/85.0)	^152^
PC	70	66 (58–73)	n.a.	Blood cfDNA mutations	KRAS	Chemotherapy	Benefit regarding OS and PFS of patients pretherapeutic ctDNA positive and turned ctDNA negative during chemotherapy	^153^
PC	47	66 (27–78)	n.a.	Blood cfDNA mutations	KRAS	Chemotherapy	KRAS mutation disappeared during the duration of stable disease or a partial response, and reappeared at the time of progressive disease	^154^
GC	136	59 (25-80)	n.a.	Blood CTCs	DAPI+/CD45–/CK+ cells	Chemotherapy	Conversion to a favourable CTC level following therapy improved the prognosis	^155^
GC	136	≤59 >59	30 29	Blood CTCs	DAPI+/CD45–/CK+ cells	Chemotherapy	Low baseline CTC count or a decrease in the CTC count after the first cycle of chemotherapy may benefit significantly from palliative chemotherapy	^156^
BTC	349	63 (54–69)	n.a.	Blood cfDNA mutations	FGFR2, IDH1,BRAF V600E	Targeted therapy	Identifying patients who may benefit from targeted therapy	^157^
BTC	124	65 (28–87)	n.a.	Blood cfDNA mutations	BRAF, ERBB2, FGFR2,IDH1	Targeted therapy	Identifying patients who may benefit from targeted therapy	^42^
NSCLC	150	68 (33–91)	n.a.	Blood ctDNA	NGS assay targeting 37 genes	Chemotherapy Targeted therapy	The use of plasma ctDNA genotyping before tissue diagnosis was associated with accelerated time to treatment	^158^
NSCLC	904	61 (28–94)	n.a.	Blood ctDNA	NGS assay targeting 101 genes	Chemotherapy Targeted therapy	The use of ctDNA testing as a reliable and complementary method to traditional tissue-based molecular analysis, enhancing the precision of treatment strategies	^159^
NSCLC	160	≤65 >65	24 136	Blood ctDNA	METex14	Targeted therapy	The broader adoption of liquid biopsy testing for identifying patients with METex14 NSCLC who do not have adequate tumour samples for biomarker testing or are ineligible for invasive tissue biopsy	^160^
GIST	45	59 (24–81)	n.a.	Blood ctDNA mutations	KIT exon 11	Targeted therapy	Ponatinib demonstrated better activity in KIT exon 11-positive GIST	^161^
CRC	294	≤70 >70	207 87	Blood ctDNA	Blood ctDNA positive or negative	Adjuvant chemotherapy	A ctDNA-guided approach reduced adjuvant chemotherapy use without compromising RFS.	^72^
CRC	62	≤65 >65	31 31	Blood ctDNA mutations	RAS, BRAF	Targeted therapy	Patients with plasma *RAS/BRAF* WT ctDNA had better outcomes	^162^
CRC	93	61 (26–80)	n.a.	Blood ctDNA mutations	16 targeted gene panel	Targeted therapy	Changes in ctDNA mutation show a significant correlation with tumour response	^163^
CRC	117	65 (10.4)	n.a.	Blood cfDNA mutations	RAS, BRAF and EGRF mutations	Targeted therapy	The dynamics of the genomic landscape in ctDNA may provide relevant information for the management of mCRC patients	^164^
CRC	100	65 (50)	n.a.	Blood cfDNA mutations	KRAS/NRAS/BRAF	Targeted therapy	75% of patients showed concordant results between liquid biopsy and tissue biopsy	^165^
Endometrial cancer	61	66.9 (60.6–73.3)	n.a.	Blood cfDNA mutations	Repair/microsatellite instability and TP53 gene mutant	Targeted therapy	Patients received a molecularly matched therapy, and presented with a 56% response rate and a median PFS of 7.7 months	^166^
ER+/HER2− Breast Cancer	172	61 (50–70)	n.a.	Blood ctDNA mutations	ESR1	Endocrine therapy	In the setting of an ESR1 mutation rising, a switch of endocrine therapy improved PFS	^167^
Breast cancer	8	50 (48–57)	n.a.	Blood ctDNA mutations	Blood ctDNA mutations	Endocrine therapy	Dynamic changes in ctDNA were observed in short timescales between treatments and support the clinical benefit seen in individual patients and appear informative of acquired resistance in real time	^168^

These presented clinical studies focus on the application of liquid biopsy as a basis for guiding treatment, enabling patients to receive personalized precision therapy.

*HCC* hepatocellular carcinoma, *NSCLC* non-small cell lung cancer, *PC* pancreatic cancer, *BC* bladder cancer, *GC* gastric cancer, *CRC* colorectal cancer, *BTC* biliary tract cancer, *GIST* gastrointestinal stromal tumour.

Therapeutic interventions tailored to ctDNA alterations demonstrated a significant, independent correlation with enhanced treatment responses or survival outcomes in patients with biliary tract cancers^70^ and gynaecological cancers^71^. On the other hand, we cannot overlook concerns regarding molecular test-based strategies that may result in either overtreatment or undertreatment. A ctDNA-guided approach to the treatment of stage II colon cancer reduced adjuvant chemotherapy use without compromising RFS^72^. However, some researchers have raised concerns that despite fewer patients receiving chemotherapy in the ctDNA group, a higher proportion of them were treated with oxaliplatin, potentially impairing their quality of life^73^.

Also, age-related mutations and epigenetic alterations should not be overlooked when interpreting liquid biopsy results. Liquid biopsies can detect polyclonal mutations, and, in older adults, tumours often exhibit both polyclonal mutations and distinct DNA methylation patterns, which may influence therapeutic response and prognosis^74,75^. In particular, PIK3CA and CDH1 mutations are more prevalent in older individuals, especially in invasive lobular breast cancer, whereas the BRAF V600E mutation is frequently observed in older adults with colorectal cancer, particularly in microsatellite instability-high tumours^76^. These findings underscore the need to account for age-related molecular features when designing and interpreting liquid biopsy assays, thereby improving the precision and personalization of cancer care for older adults.

### Potential applications of liquid biopsies in response monitoring and prognostication

In older adults, CHIP-related mutations present in cfDNA may be misinterpreted as tumour-derived ctDNA, as CHIP is more prevalent with increasing age^77^. Nevertheless, several emerging validation approaches can help improve discrimination. For instance, fragment length analysis has shown that tumour-derived cfDNA typically displays a shorter fragment size distribution compared with CHIP-derived cfDNA^78^. Likewise, tumour fractions estimated from cfDNA methylation profiles have demonstrated a sensitivity of 86.1% for early cancer detection and an average accuracy of 76.9% for tumour localization at a specificity of 94.7%^79^. Additional methods, including nucleosome positioning frameworks^80^ and machine-learning models using genome-wide mutational profiles^81^ further enhance the ability to distinguish true tumour-derived signals from cfDNA variants arising from clonal haematopoiesis and other sources of background noise, thereby mitigating the confounding effect of CHIP. Liquid biopsies are less invasive than tissue biopsies, making them a very attractive option in the older population, or in people with multiple comorbidities^77^. This aspect is particularly important in the older patient population, where we can frequently encounter difficulties in obtaining tissue biopsies (such as anticoagulation, positioning difficulties, and transportation, among others).

Liquid biopsies may yield significant advantages for treatment response monitoring purposes in older patients compared with standard tissue biopsies (Table 3). Liquid biopsies are cheaper, less invasive, and can anticipate the evidence of radiological disease progression^82^. They may also enable the detection of tumour shedding from multiple metastatic lesions rather than individual deposits (as with tissue biopsies) and therefore overcome the challenges of addressing tumour heterogeneity. On the other hand, several technical difficulties have limited standardization and acquisition of these approaches^77^.

**Table 3:** Liquid Biopsy for Tumour Response Monitoring and Prognosis

Tumour	Patient population	Age group	Number of patients in the corresponding age group	Format	Target	Clinical significance	Primary Outcomes	Reference
HCC	344	≤50 >50	141 203	Blood CTCs	DAPI+/CD45–/CK+ cells or DAPI+/CD45–/EpCAM+ cells	CTC-positive patients benefit more from TACE HR = 0.46 (TTR) HR = 0.32 (OS)	Recurrence and survival	^169^
HCC	87	63 (60–71)	n.a.	Blood CTCs	DAPI+/CD45–/CK+/PDL1+ cells	PD-L1+CTCs predicted shorter OS HR = 3.22	HCC detection and prognostication	^170^
HCC	270	55 (31–78)	n.a.	Blood CTCs	DAPI+/CD45–/CK+ cells	CTC number predicted recurrence HR = 11.89 CTC clusters showed a prediction ability for recurrence HR = 13.67	Recurrence	^171^
HCC	227	≤45 >45	103 125	Blood CTCs	CanPatrol^TM^ CTC-enrichment technique	CTC number predicted recurrence HR = 1.98	Recurrence	^172^
HCC	60	≤60 >60	16 44	Blood extracellular vesicles	EV-cfDNA to detect c.747G>T mutation in the TP53 gene	Patients with high-frequency mutation had shorted RFS HR = 4.61	Recurrence	^173^
HCC	258	54	n.a.	Blood cfDNA mutations	ARID1A, CDKN2A, FAT1, LRP1B, MAP3K1, PREX2, TERT and TP53	Patients with high-risk genes had shorter RFS HR = 7.1	Recurrence	^174^
HCC	498	≤50 >50	122 376	Blood ctDNA methylation signatures	Blood ctDNA methylation signatures	High differentially methylated regions score showed a higher recurrence risk HR = 3.33 (RFS)	HCC detection and prognostication	^175^
NSCLC	46	63 (58–70)	n.a.	Blood ctDNA	Baseline summatory mutant allelic fraction	Higher baseline summatory mutant allelic fraction is associated with shorter OS and PFS HR = 4.38 (OS) HR = 3.26 (PFS)	Recurrence and Survival	^176^
NSCLC	25	≤65 >65	21 4	Blood ctDNA	Tumour mutation burden	Higher tumour mutation burden is associated with shorter OS and PFS HR = 0.062 (OS) HR = 0.197 (PFS)	Recurrence and survival	^177^
PC	40	≤50 >50	13 27	Blood CTCs	CK^+^/CD45−/ KLF8^+^ cells or CK^+^/CD45−/ vimentin^+^ cells	Patients had a higher relapse rate	Recurrence	^178^
PC	60	64.6 (27–88)	n.a.	Blood CTCs	ALDH-positive CTC	CTCs are independently predictive of decreased DFS and OS HR = 3.4 (recurrence)	Recurrence and survival	^179^
PC	50	64.9 (27–86)	n.a.	Blood CTCs	DAPI+/CD45–/CK+ cells	The detection of CTCs was predictive of recurrence HR = 2.78 (recurrence)	Recurrence and survival	^180^
PC	36	64 (52–73)	n.a.	Blood CTCs	DAPI+/CD45–/CK+ cells or DAPI+/CD45–/EpCAM+ cells	CTCs positivity was an independent risk factor for recurrence OR = 8.77 (early recurrence) R = 5.6 (systemic recurrence)	Recurrence	^181^
PC	n.a.	n.a.	n.a.	Blood cfDNA mutations	KRAS	Patients with G12D KRAS mutations had shorter OS HR = 1.417 (OS)	PC detection and prognostication	^182^
PC	103	≤67 >67	51 52	Blood ctDNA methylation signatures	KRAS	KRAS status was an independent unfavourable prognostic factor for shorter OS and RFS HR = 4.9–6.9 (OS) HR = 3.4–3.7 (RFS)	Recurrence and survival	^183^
PC	145	71 (50–86)	n.a.	Blood cfDNA mutations	KRAS, TP53	KRAS or TP53 mutations predict shorter RFS	Recurrence	^184^
PC	61	65 (40–84)	n.a.	Blood ctDNA mutations	KRAS	KRAS status was an independent unfavourable prognostic factor for shorter OS and PFS HR = 5.692 (OS) HR = 8.631 (PFS)	Progression and survival	^185^
PC	104	63 (36–86)	n.a.	Blood ctDNA and exoDNA	KRAS	Detection of ctDNA was associated with worse OS and PFS HR = 2.36 (OS) HR = 1.93 (PFS) ExoKRAS ≥ 5% was associated with worse OS and PFS HR = 7.31 (OS) HR = 4.38 (PFS)	Progression and survival	^186^
CRC	287	64 (27–96)	n.a.	Blood CTCs	CellSearch system	CTC was significantly associated with worse OS and PFS Stage I–III: HR = 5.5 (OS) HR = 12.7 (PFS) Stage I–IV: HR = 5.6 (OS) HR = 7.8 (PFS)	Progression and survival	^187^
CRC	493	62 (24–75)	n.a.	Blood cfDNA	Blood cfDNA	High cfDNA levels were associated with impaired outcome HR = 1.83 (OS) HR = 1.43 (PFS)	Progression and survival	^188^
CRC	148	≤70 >70	105 30	Blood ctDNA mutations	Variant allele fraction analysed by means of a 14-gene NGS panel	Patients with high ctDNA VAF had poorer OS HR = 1.82 (OS)	Survival	^189^
CRC	299	≤70 >70	248 51	Blood ctDNA methylation signatures	BCAN, BCAT1, IKZF1, Septin9_1, Septin9_2, VAV3	ctDNA-positive patients had poorer RFS HR = 17.5 (Recurrence) HR = 13.8 (RFS)	Progression and survival	^190^
GC	148	≤65 >65	75 73	Blood ctDNA methylation signatures	SFRP2	The top 50% methylated SFRP2 had shorter PFS and OS Stage III: HR = 7.8 (OS) HR = 13.05 (PFS) Stage IV: HR = 3.14 (OS) HR = 2.74 (PFS)	Progression and survival	^191^
HR+/HER2− breast cancer	36	59.5 (37–79)	n.a.	Blood ctDNA mutations	PIK3CA	An activating PIK3CA mutation at baseline was associated with a shorter PFS HR = 4.4 (PFS)	Progression	^192^
Glioblastoma	19	52 (21–72)	n.a.	Blood ctDNA	Blood ctDNA maximal variant allele frequency (MVAF)	Patients with a reduction in MVAF after treatment exhibited extended median OS HR = 0.21 (OS)	Progression and survival	^193^
Melanoma	69	≤60 >60	35 34	Blood ctDNA	Blood cfDNA clearance after treatment	Patients manifesting a 90% or greater reduction in ctDNA after treatment demonstrated markedly prolonged OS and PFS HR = 49.6 (OS) HR = 5.8 (PFS)	Progression and survival	^194^

These presented clinical studies focus on developing the prognostic value of liquid biopsy to identify patients at high risk of recurrence/progression, enabling potential interventions.

*HCC* hepatocellular carcinoma, *NSCL* non-small cell lung cancer, *PC* pancreatic cancer, *BC* bladder cancer, *GC* gastric cancer, *CRC* colorectal cancer.

Liquid biopsies may also facilitate monitoring the tumour response to ongoing treatments. In patients with breast cancer, liquid biopsies predict the risk of metastatic recurrence after surgery ~8 months before there is any radiological evidence of metastases^83^. Similar findings have been documented in lung, colorectal, and pancreatic cancer after initial treatment^82,84^.

Liquid biopsies may facilitate early detection of disease recurrence in specific lung cancers, as well as pancreatic cancer, colorectal cancer, and melanoma with specific mutations^82,84^. The use of liquid biopsies for monitoring of patients with metastatic ovarian and breast cancers has also shown feasibility^82^. Finally, liquid biopsies may detect the presence of alterations associated with the development of treatment resistance and inform the selection of specific treatments yielding better chances of response^85^.

Nonetheless, more research is needed to standardize the tests and validate these hypotheses across various tumour types in older adults. Regardless of whether patients with cancer undergo curative-intent surgery or palliative systemic therapy, long-term and regular follow-up is essential.

Though for older patients with limited mobility, the collection of medical history and blood samples can be conducted with relative ease, imaging assessments remain indispensable. If potential correlations between liquid biopsy data and imaging findings can be identified, such that the frequency of imaging follow-up can be appropriately reduced, it may significantly improve the quality of life and alleviate economic burden during the follow-up process.

### Conclusions and future perspectives

Considering the age distribution of participants in any liquid biopsy trial is key to ensuring the translatability of results to older adults. Recruitment into some major liquid biopsy trials is encouraging; for example, initial results from the GALAXY trial (part of the CIRCULATE-Japan study reviewing ctDNA-based molecular residual disease detection and adjuvant chemotherapy efficacy in CRC) show good recruitment in older adults with a median age of 69 years, maximum age of 93 years, and 43% of the 1039-patient cohort were aged >70^86^.

Before liquid biopsies can be widely implemented in routine clinical care for older adults, several challenges must be addressed. First, considerable work is required to standardize the pre-analytical, analytical, and post-analytical phases. In the case of ctDNA, this is well described in a special report from the European Society for Medical Oncology (ESMO) Precision Cancer Working Group^38^. However, similar guidelines are not available for other liquid biopsy approaches. As technology progresses and translates into clinical practice, such guidelines will be crucial to ensuring patient safety. Socioeconomic costs are another concern, as high-quality studies often rely on deep sequencing and multi-omics approaches^87^, which improve evidence quality and understanding of aging biology but increase the burden on healthcare systems^88^. Practical issues, including sampling difficulties in frail older adults and the risk of overdiagnosis, may not be apparent in research settings but could arise in real-world practice.

In recent years, there has been a burgeoning trend in the pharmaceutical industry towards incorporating the analysis of ctDNA or cfDNA as biomarkers in clinical trial protocols for a spectrum of oncological drugs (Table 4). These include targeted therapy drugs^89–91^, immune checkpoint inhibitors^92,93^, and antibody-drug conjugates^94,95^. The inclusion of ctDNA and cfDNA as predictive or prognostic biomarkers is becoming standard practice, particularly in the context of malignancies prevalent in the older population, such as NSCLC, breast cancer, and gastrointestinal malignancies (Table 2).

**Table 4:** Applications of liquid biopsy in oncology clinical trials

Tumour	Setting	Types of liquid biopsy	Treatment	No. of patients	No. of patients ≥65 years (%)	Median/mean age (IQR/range/SD)	Key outcomes in older patients	Reference
Melanoma	Adjuvant	cfDNA	mRNA-4157 (V940) plus pembrolizumab	157	70 (44.6%)	–	RFS HR: 0.979(0.413–2.318)	^195^
	Palliative 1st line	ctDNA	Tebentafusp	252	122 (48.4%)	Median 64.0 (range 23–92)	OS HR: 0.84(0.60–1.19)	^196^
NSCLC	Neoadjuvant	ctDNA	Nivolumab	86	39 (45.3%)	–	OS HR: 0.64(0.16–2.51) PFS HR: 0.42(0.17–1.06)	^93^
	Palliative 1st line	cfDNA	Ramucirumab plus erlotinib	449	233 (51.9)	–	PFS HR: 0.77 (0.55–1.09)	^89^
	Palliative 2nd line	cfDNA	Tepotinib plus osimertinib	128 (98 analysis)	31 (31.6%)	Median 61.0 (IQR 52–67)	ORR 58.1% (39.1–75.5)	^90^
		cfDNA	Tepotinib	99	≥75 years 45 (45.5%)	Median 74.0 (Range 41–94)	≥75 years ORR 42.2% (27.7–57.8)	^91^
Pleural mesothelioma	Palliative 1st line	cfDNA	Anetumab ravtansine	248	145 (58.5%)	–	PFS HR: 1.28(0.79–2.08)	^94^
Anal cance**r**	Palliative 1st line	ctDNA	Atezolizumab plus modified docetaxel, cisplatin, and fluorouracil	100 (97 analysis)	44 (45.4%)	–	PFS HR: 0.843(0.394–1.801)	^92^
Breast cancer	Palliative 2nd line	ctDNA	Buparlisib plus fulvestrant	432	158 (36.6%)	–	PFS HR: 0.843(0.394–1.801)	^197^
Prostate cancer	Palliative 1st line	ctDNA	Olaparib plus abiraterone	796	569 (71.5%)	–	OS HR: 0.95(0.75–1.19)	^198^
		ctDNA	Talazoparib plus enzalutamide	805	≥70 years 480 (59.6%)	–	≥70 years PFS HR: 0.67(0.51–0.89)	^199^
GC/EAC	Palliative 1st line	ctDNA	Bemarituzumab	155	44 (28.4%)	–	OS HR: 0.82(0.35–1.91) PFS HR: 0.71(0.33–1.56)	^200^
	Palliative 2nd line	ctDNA	Margetuximab plus pembrolizumab	95	40 (42.1%)	Median: 61.0 (IQR: 52.0–70.0)	OS HR: 0.68(0.39–1.18) PFS HR: 0.50(0.31–0.80)	^201^
CRC	Palliative 2nd line	cfDNA	Trastuzumab deruxtecan	78 (53 analysis)	18 (34.0%)	Median 57.0 (IQR 50.0–66.0)	ORR 50.0% (26–74)	^95^
BTC	Palliative 2nd line	ctDNA	Futibatinib	103	23 (22.3%)	Median 58.0 (range 22–79)	ORR 50.0% (26–74)	^202^
Ovarian cancer	Palliative 2nd line	ctDNA	Niraparib and anlotinib	40	≥55 years 18 (35.0%)	Median 54.0 (range 37–69)	≥55 years PFS HR: 1.25(0.34–3.92)	^203^

These presented clinical studies focus on the treatment of malignant tumours and highlight the therapeutic effects in older adults as a subgroup. While liquid biopsy is not the primary endpoint of these studies, its role as an exploratory endpoint suggests it may hold greater potential for future applications.

*NSCLC* non-small cell lung cancer, *GC* gastric cancer, *EAC* esophageal adenocarcinoma, *CRC* colorectal cancer, *BTC* biliary tract cancer.

Our work has certain limitations. First, apart from MCED studies, most research has not been specifically designed for older populations, resulting in limited representation of older adults. In addition, there is considerable heterogeneity among studies, including differences in patient inclusion criteria, assay platforms, sample processing, and model construction. Moreover, due to the broad temporal and geographical span of these studies, some early small-sample data may not be generalizable.

With an estimated 1.6 billion people over 65 by 2050^96^, there will be a significant rise in age-related diseases, including cancer. Therefore, it is crucial to elucidate the interplay between ageing and cancer and develop strategies for early detection, prevention, and personalized interventions in this rapidly expanding population. Importantly, chronological age alone offers an incomplete representation of an individual’s physiological reserve and overall health status^97,98^. A more informative estimation of biological age, incorporating functional, molecular, psychological, and social dimensions, is critical for guiding therapeutic decision-making in older adults with cancer^99^.

Biological age influences not only treatment tolerance and expected benefit, but also a patient’s readiness and engagement throughout the course of medical care^100^. CGA remains one of the most established clinical approaches for capturing functional and health-related components of biological age. However, its implementation can be limited by variability across assessment domains and the reliance on measures that may fluctuate with clinical context. In parallel, liquid biopsy technologies offer relatively consistent, tumour- and aging-related molecular insights, especially when monitored over time. Integrating CGA with liquid biopsy data may improve biological age estimation, risk stratification, and individualized management. Accordingly, evaluating the feasibility and performance of liquid biopsy in older populations is an important research priority. Clarifying the convergence of clinical and molecular aging markers may ultimately enhance person-centred cancer care in older adults.

## Data Availability

No datasets were generated or analysed during the current study.
